# Unexpected discovery of hemoglobinopathy C/β° thalassemia

**DOI:** 10.1002/ccr3.1815

**Published:** 2018-09-21

**Authors:** Wafaa Bouyarmane, Jean Uwingabiye, Asmaa Biaz, Achraf Rachid, Youness Mechal, Abdellah Dami, Sanae Bouhsain, Zhor Ouzzif, Samira El Machtani Idrissi

**Affiliations:** ^1^ Department of Clinical Biochemistry and Toxicology Mohammed V Military Teaching Hospital Faculty of Medicine and Pharmacy Mohammed V University Rabat Morocco

**Keywords:** β° thalassemia, double heterozygosity, hemoglobinopathy C, high performance liquid chromatography

## Abstract

High performance liquid chromatography (HPLC) is the current method of choice for the detection of hemoglobinopathies and the quantification of A2 and fetal hemoglobin. We are describing a case where a double heterozygosity C/beta‐thalassemia was fortuitously identified, during assaying HBA1c, by HPLC.

## 1 INTRODUCTION

Hereditary disorders of hemoglobin are probably the most common genetic abnormalities concerning either structural defects in the hemoglobin molecule or alterations of globin chain synthesis. Beta‐thalassemia is an alteration that, in the heterozygous (asymptomatic) state is mostly easily identifiable by an increase in the A2 fraction of the hemoglobin molecule. Homozygous beta‐thalassemia (thalassemia major or Cooley's anemia) is a serious disease that imposes a large change in quality of life (transfusion, chelating, etc.) to allow the patient to survive into adulthood, curable by a bone marrow transplant. C/beta‐thalassemia hemoglobinopathy (HbC‐BT) is a form that may be related to beta‐thalassemia intermediate or homozygous hemoglobin C according to whether it is the β° or β+ allele. Clinical manifestations include moderate to severe anemia and splenomegaly. We are reporting a case unexpected discovery of C/β°‐thalassemia hemoglobinopathy in adulthood, following the assay of HbA1c by HPLC.

## 2 CASE REPORT

A 53‐year‐old male Moroccan patient who was followed for diabetes outside of health facility, consulted for a hot thyroid nodule of recent appearance. In the context of an extensive assessment, HbA1c assay was requested, it was performed in a private laboratory and objected to the absence of HbA1 with the presence of a hemoglobin C variant. The diagnosis of a homozygous hemoglobin C disease was retained, and the determination of HbA1c was impossible. The patient is then sent to our laboratory to explore his hemoglobinopathy.

**Figure 1:**
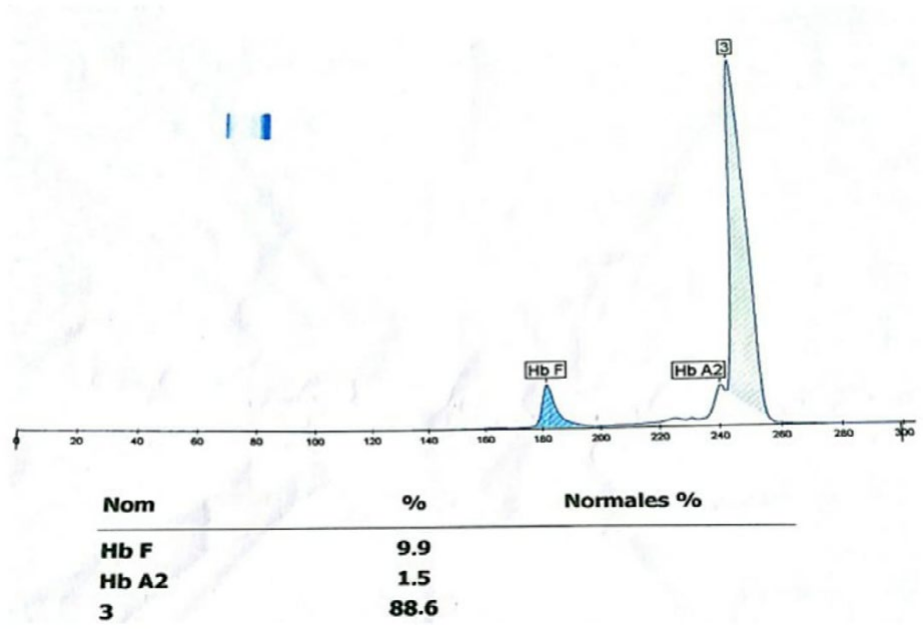
Capillary electrophoresis of hemoglobin

**Figure 2:**
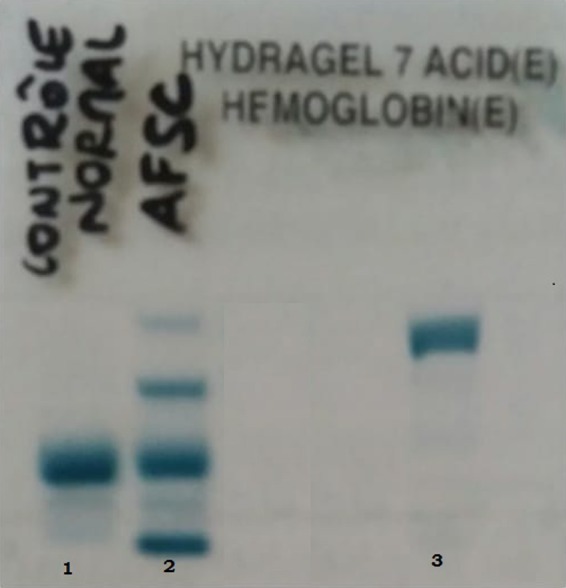
Acidic hemoglobin electrophoresis, lane 1 = normal control, lane 2 = pathological control AFSC and lane 3 = patient profile (hemoglobin C)

Capillary electrophoresis in alkaline buffer (pH 9.4) with the SEBIA CAPILLARYS 2 showed a peak migrating at zone 250 with a rate equal to 88.6%, the rate of HbF and HbA2 were 9.9% and 1.5%, respectively (Figure 1). Hemoglobin electrophoresis showed a variant located in zone 3 corresponding to the migration zone of HbC; an acidic pH electrophoresis stained with amidoschwartz on Sebia Hydrasys was necessary and showed an abnormal band migrating upstream of the area of the deposit corresponding to variant C of Hb (Figure 2). Confirmation by high performance liquid chromatography (HPLC) on D‐10 automaton (Bio‐Rad^®^ Biorad Diamat; Biorad, Ivry Sur Seine, France) in Varian © mode was performed to quantify the fractions F and A2 of hemoglobin (HbF: 5.8% and HbA2: 5%).

With the HbF value ranging between 2% and 10% and the HbA2 > 3.4%, we suspected a combination of beta‐thalassemia. The patient's blood samples were then sent for genotypic study. Betag lobin sequencing on Applied 3130XL showed the presence of two mutations in the heterozygous state: HbC‐HBB: c.19G>A and Cd6 (‐A)‐HBB: c.20delA.

The hemogram revealed a discrete anemia (Hemoglobin = 11.5 g/dL), microcytosis (Mean corpuscular volume at 65 fL), hypochromia (Mean corpuscular hemoglobin at 20 pg), pseudoglobulia (red blood cells at 5.68 × 10^6^/μL), the hematocrit was slightly low (37%), and thrombocytopenia was noted (83 000/mm^3^). The blood smear showed erythrocyte anisopoikilocytosis with microcytes and Target cells.

A biochemical test was carried out: The blood ionogram, the hepatic and lipid assessment were normal, however an increase of the total bilirubin and the indirect bilirubin, the ferritin and the iron are slightly above the normal, the TSHus as well as haptoglobin were decreased (Table 1). The biological assessment confirmed the hemolytic anemia. This patient has no surgical or medical history apart from jaundice and skin lesions due to psoriasis. Clinical examination revealed moderate and isolated splenomegaly. No particular family history, no notion of hemoglobinopathy in the siblings. No parental consanguinity. The family survey could not be carried out because of death of both parents.

**Table 1:** Biochemical assessment

Parameters	Results	Reference range
Sodium	140 mmol/L	136‐144
Potassium	4.4 mmol/L	3.6‐5.1
Chlorine	106 mmol/L	101‐111
Bicarbonate	27.00 mmol/L	22‐32
Total protein	71 g/L	60‐80
Urea	0.25 g/L	0.17‐0.43
Glycemia	1.13 g/L	0.70‐1.05
Creatinine	6 mg/L	6‐13
Uric acid	65 mg/L	39‐78
Total bilirubin	29 mg/L	3‐12
Direct bilirubin	4 mg/L	1‐5
AST	17 U/L	15‐41
ALT	25 U/L	14‐54
PAL	54 U/L	32‐91
GGT	34 U/L	7‐50
LDH	109 U/L	98‐192
CHOLESTEROL	0.75 g/L	1.50‐2.00
Triglyceride	0.72 g/L	0.60‐1.50
CRP	1 mg/L	1.0‐7.5
Iron	190 μg/dL	45.00‐182.00
Ferritin	487 ng/mL	11.0‐336.00
TSH us	0.07 μU/mL	0.34‐5.60
Haptoglobin	<0.287 g/L	0.3‐2

## 3 DISCUSSION

Hemoglobinopathies are either quantitative abnormalities, such as thalassemias, or qualitative abnormalities that lead to abnormal Hb. More than 1600 molecular anomalies of globin genes are identified, including more than 800 of the β chain.^1^, ^2^ New mutations are regularly discovered, expanding this list of these anomalies. These genes are located on chromosomes 11 and 16. The consequences of alterations are abnormal Hb or total or partial synthesis reduction. Both types of anomalies can be associated. Beta‐thalassemia is a group of heterogeneous disorders of autosomal recessive inheritance. They are endemic in the Mediterranean basin but also highly present in West Africa and South Asia.^3^, ^4^ They are due to point mutations, microdeletions or insertions of nucleotides.^5^ β‐thalassemia is the consequence of the reduced (β+) or absent (β°) synthesis of the beta globin chain, resulting in an excess of residual alpha chains that precipitate in the precursor RBC leading to premature destruction in the bone marrow. This inefficient erythropoiesis adds up to the peripheral hemolysis which is due to the alteration of the RBC membrane by α chain precipitation at this level, in addition to a decrease in the synthesis of Hb, it results in a severe anemia. β‐thalassemias are divided into three major types according to the importance of the clinical signs expressed^6^, ^7^: β‐thal major or Cooley anemia; Intermediate β‐thal and minor β‐thal. However, there are many variations depending on the variety and the impact of mutations observed. The severity of the clinical symptoms is directly proportional to the importance of the mutations. Hemoglobin C is common in West Africa.^8^ It is an abnormal hemoglobin that reflects the mutation of the sixth amino acid of the β‐globin chain^5^; this mutation concerns the first nucleotide of this codon: it is the substitution of guanine by adenine (GAG → AAG). This results in the replacement of glutamic acid with lysine (β6glu → lys).

In the C/β‐thal composite forms, the subject inherits a thalassemic trait from one of his parents and an abnormality responsible for the hemoglobinosis of the other. It is characterized by microcytosis, hypochromia and pseudopolyglobulia.^8^ This heterozygous association is expressed in two modes: C/β° in which the HbA is absent and the C/β+ with aHbA level which can reach 30%. Β+ is more common than β° in subjects with HbC; this composite heterozygosity is observed more particularly in people of African descent, and it is also reported in North Africa, Italy (Sicily) and Turkey.^9^ The clinical picture of Hemoglobin C/beta‐thalassemia is similar to that of a Hemoglobin CC disease.^10^ According to the nomenclature of acts of medical biology, the search for an anomaly of the hemoglobin is done, by at least one electrophoretic technique and two other tests adapted according to the need for a diagnostic result of orientation (Act 1120, B120).^11^ Among the three techniques used, one must be quantitative in order to accurately measure HbA2 and HbF. Only high performance cation exchange liquid chromatography (HPLC) and capillary electrophoresis (ECAP) are used for quantification and are fully automated. In the event of a fortuitous discovery of aHb anomaly during the assay of HbA1c by HPLC, which is the case here, confirmation by electrophoresis at alkaline and/or acidic pH is necessary^12^, ^13^, ^14^.

The high HbA2 level is explained by a constitutional increase due to β‐thalassemia by union of alpha chains in excess to the delta chains.^5^ The difference in HbA2 levels between capillary electrophoresis (1.5%) and HPLC (5%) can be explained by the fact that there are no standardized techniques for the determination of HbA, we measured the HbA2 level by capillary electrophoresis and HPLC by using two different samples, and the HbA2 level is uninterpretable in the presence of variant. The acquired increase in the expression of HbA2 is rarely observed during untreated hyperthyroidism, in megaloblastic anemias or in HIV patients treated with antivirals.^15^

The elevation of HbF would play a protective role by inhibiting the crystallization of HbC.^8^ Two mutations were detected in our patient: HBB: c.19G>A corresponding to Hb C and the Cd6 mutation (‐A; HGVS: HBB: c.20delA) responsible for β‐thalassemia. It is positioned on exon 1.^16^ Depending on the number of amino acids affected and the location of the mutation on the gene, this may lead to different clinical consequences. Point mutations are largely the most frequent (there are nine point mutations, deletions or short insertion for one wide deletion). When point mutations are located at the level of the promoter or introns, they usually have fewer consequences than mutations at splice sites or mutations affecting a large part of the gene. The Cd6 (‐A) mutation of Mediterranean origin is responsible for the β° phenotype.^17^ In a typical way, each of the regions of the globe presents a spectrum of mutations of its own.^18^ Fattoum reports in his study in 2006, 19 mutations of β‐thalassemia including two predominant mutations, namely, Cd 39C → T and IVSI‐110G → A.^19^ The study of codon 39 and IVS‐I‐110 haplotypes links their origins to North Africa and the eastern Mediterranean, respectively.^20^ In a study conducted in Buenos Aires, the Cd6 (‐A) mutation was found in 6 out of 483 patients included in the study, including one patient with thalassemia major. The objective mutations in C/β‐thalassemia double heterozygotes are Cd 39 C → T and IVSI‐110 G → A.^16^ Cd6 (‐A) also known as FSC 6 reaches its highest frequency in Algeria with 17.7% followed by Morocco with 10% of β‐thalassemic chromosomes study.^21^, ^22^ It is associated with two haplotypes III and IX,^4^ which are also common in Morocco and Algeria.

## 4 CONCLUSION

Our observation points out two important points: The HbA1c test should not be done by immunological technic in a patient whose hemoglobin status is not known; separation techniques must be performed. The differential diagnosis between homozygous hemoglobin C and double heterozygosity C/β°‐thalassemia requires the use of a family survey associated with genotyping, to better detect people at risk and offer genetic counseling.

## CONFLICT OF INTEREST

None declared.

## AUTHOR CONTRIBUTIONS

UJ, WB and SE drafted the initial manuscript, assisted with revision process and collected photographs and data to be used in manuscript. AB, AR, YM, AD, SB, ZO advised on the discussion and revised the manuscript.
